# Embryonal Rhabdomyosarcoma of the Adult Urinary Bladder: A Rare Case Report of Misclassification as Inflammatory Myofibroblastic Tumor

**DOI:** 10.1155/2015/510508

**Published:** 2015-02-09

**Authors:** Kelven Weijing Chen, Fiona Mei Wen Wu, Victor Kwan Min Lee, Kesavan Esuvaranathan

**Affiliations:** ^1^Department of Urology, National University Health System, 1E Kent Ridge Road, Singapore 119228; ^2^Department of Pathology, National University Health System, 1E Kent Ridge Road, Singapore 119228

## Abstract

Embryonal rhabdomyosarcoma (ERMS) of the adult urinary bladder is a rare malignant tumour. Inflammatory myofibroblastic tumour (IMT) of the bladder is a benign genitourinary tumour that may appear variable histologically but usually lacks unequivocal malignant traits. Techniques like flow cytometry and immunohistochemistry may be used to differentiate these two tumours. Our patient, a 46-year-old male, had rapidly recurring lower urinary tract symptoms after two transurethral resections of the prostate. He subsequently underwent a transvesical prostatectomy which showed IMT on histology. However, his symptoms did not resolve and an open resection done at our institution revealed a 6 cm tumour arising from the right bladder neck. This time, histology was ERMS with diffuse anaplasia of the bladder. Rapid recurrence of urinary symptoms with prostate regrowth after surgery is unusual. Differential diagnoses of uncommon bladder malignancies should be considered if there is an inconsistent clinical course as treatment approaches are different.

## 1. Introduction

Embryonal rhabdomyosarcomas (ERMSs) are malignant soft tissue tumors that are thought to originate from immature cells destined to form striated skeletal muscle. However, these tumors can arise in locations where skeletal muscle is not typically found, for example, in the urinary bladder. In paediatric patients, 20% of rhabdomyosarcomas (RMSs) occur in the genitourinary tract and this accounts for 6% of all childhood malignancies [1]. In adults, however, ERMS of the bladder/prostate is very rare with less than 15 published case reports. As a result, optimal treatment and management of this rare but aggressive tumor can only be extrapolated from paediatric case series.

Inflammatory myofibroblastic tumors (IMTs) of the urinary tract, also termed postoperative spindle cell nodule, inflammatory pseudotumor, and pseudosarcomatous fibromyxoid tumor (PSFMT), are uncommon tumors of the genitourinary tract, with the largest series of 46 cases published by Montgomery et al. [2]. Although benign, the cellular pleomorphism and the infiltrative nature of the lesion may be mistakenly diagnosed as sarcomatoid carcinoma or sarcoma [3].

Here we describe a case of an ERMS misdiagnosed as an IMT/PSFMT. Because IMTs may resemble low-grade sarcomas and vice versa from a histologic point of view, a surgeon should always correlate clinical judgment with histopathology to reach an accurate diagnosis. This is crucial in order to prevent inadequate treatment for a malignant tumor or unnecessary radical therapy for a benign lesion.

## 2. Case Report

Mr X, a 46-year-old male, was referred from overseas to our institution for recurring lower urinary tract symptoms suggestive of benign prostate enlargement (BPE) after transurethral resection of prostate (TURP).

He initially presented at the age of 43 years with voiding symptoms such as poor stream, hesitancy, incomplete bladder emptying, and terminal dribbling. He underwent TURP in May 2010 with good resolution of his symptoms. The pathology was reported as benign nodular hyperplasia of the prostate (BPH). However, the symptoms recurred 9 months later and progressively worsened. He sought treatment from the same urologist and was diagnosed to have recurrence of his BPE. Second TURP was performed. Again, the histopathology was reported as BPH. His urinary symptoms again resolved after the second TURP but recurred just 3 months after surgery. He then underwent a transvesical prostatectomy. The histopathology was reported as PSFMT. His symptoms recurred in a similar fashion after 3 months, after which he decided to seek a second opinion at our institution.

**Figure 1:**
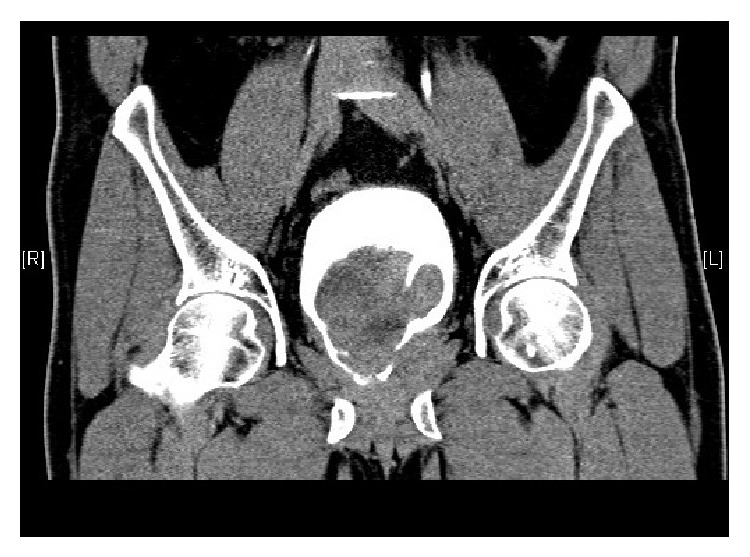


**Figure 2:**
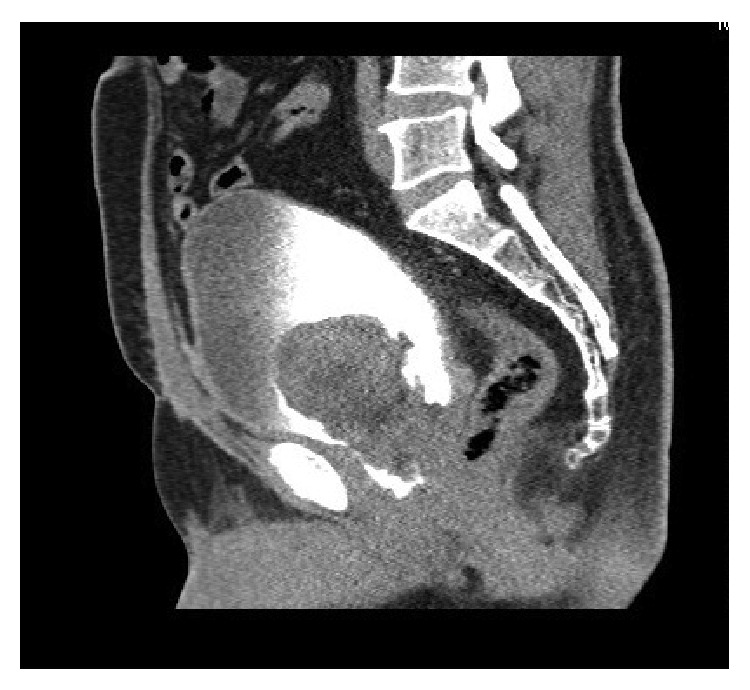


A computed tomogram of his urinary tract performed at our institution revealed a tumour arising from the base of the bladder with possible involvement of the prostate (Figures 1 and 2).

**Figure 3:**
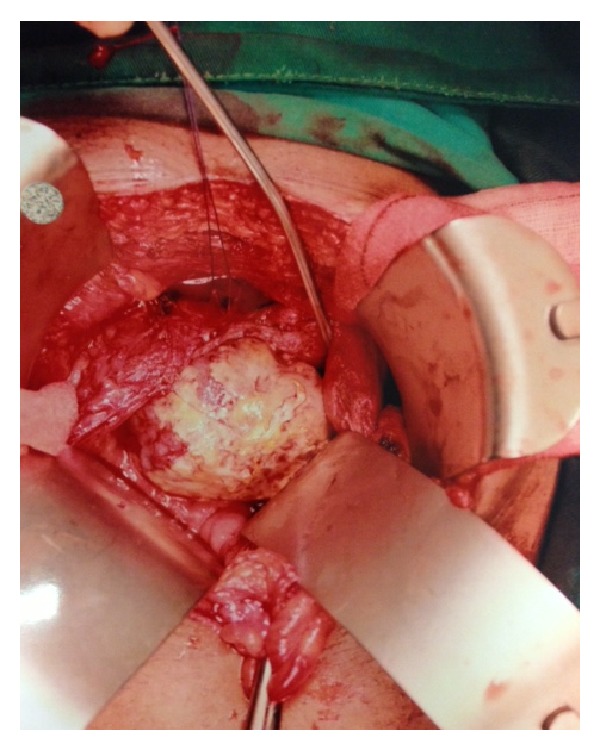


**Figure 4:**
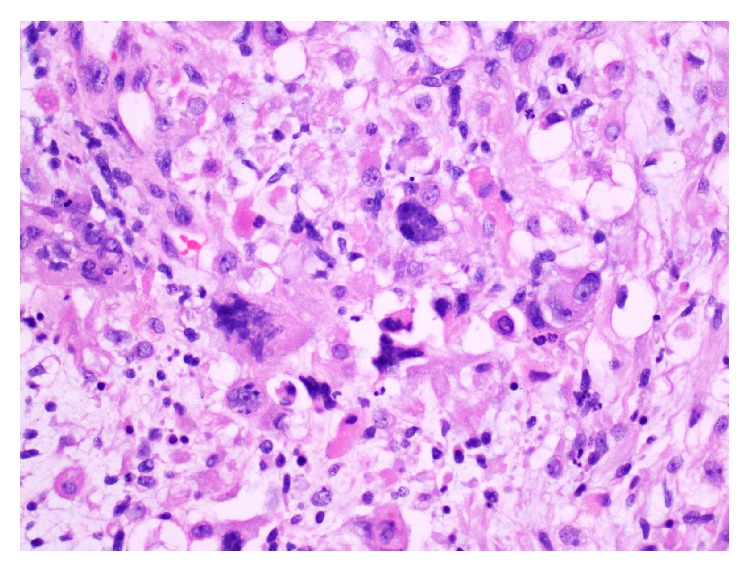


The patient underwent an open resection of his bladder/prostate tumour. A 6 cm bladder tumour (Figure 3) was found arising from the right side of the bladder neck and was traced into the right lobe of the prostate. The histology was reported as ERMS with diffuse anaplasia of the bladder and margins were negative (Figure 4).

The patient was advised to commence on chemotherapy with the standard vincristine, actinomycin-D, and cyclophosphamide (VAC) regime followed by radiotherapy, but he decided to return to his home country for further treatment.

## 3. Discussion

The histology of IMT and RMS is similar and may be confused with one another [4]. IMT is composed of widely separated spindle cells with elongated eosinophilic cytoplasmic processes that are surrounded by loose edematous stroma of mononuclear inflammatory infiltrates. The spindle cell nuclei are large, nonhyperchromatic, and variable but usually lack unequivocal malignant features. Some nuclei are prominent and resemble ganglion cells; others are eccentric and small, similar to rhabdomyoblasts [5].

Rhabdomyosarcoma, on the other hand, is composed of typical rhabdomyoblasts arranged in sheets and large nests, with infrequent intermixed fusiform cells. They contain small primitive undifferentiated cells characterized by a high nuclear-to-cytoplasmic ratio. Ro et al. [6] described 2 cases of IMT initially diagnosed to be RMS because of bizarre spindle cell proliferation, occasional mitoses, and invasion to the underlying muscle. Conversely, the diagnosis of sarcomatoid carcinoma of the bladder from PSFMT can be confusing, especially when the spindle cell component of the sarcomatoid carcinoma is myxoid and demonstrates bland cytologic features [3]. Although believed to be benign, IMT has been known to precede sarcomatoid carcinoma with high grade invasive urothelial carcinoma [2] in isolated cases. Even when the slides were reviewed again, IMT of the malignant cases cannot be differentiated from benign IMT of other cases. Hence, in atypical cases such as in our patient, the diagnosis of IMT should be made with caution.

**Figure 5:**
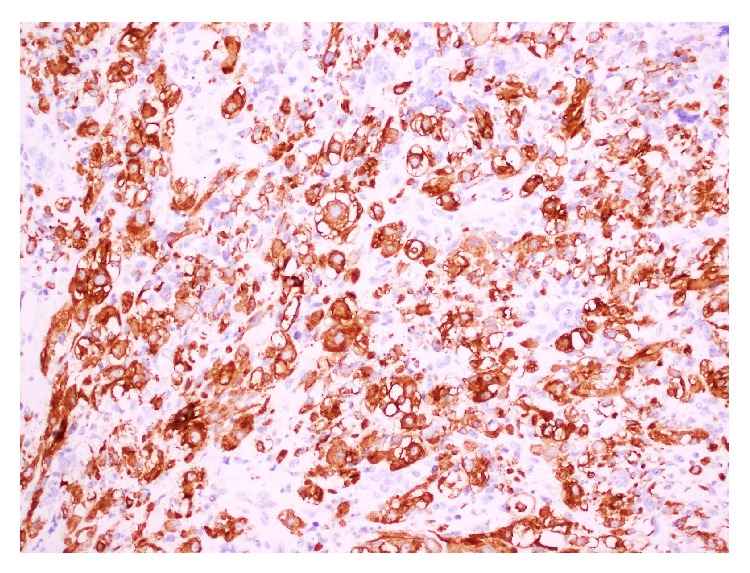


Rhabdomyosarcoma can be differentiated from IMT based on morphological features (more frequent mitoses with atypical forms and tumor-type necrosis) and the application of an immunohistochemical panel that includes MyoD1 or myogenin [7] (Figure 5). Myogenin and MyoD1 are transcriptional regulatory proteins that are both sensitive and specific for RMS. A study by Cessna et al. found all RMSs to have stained positive for MyoD1 while none of the IMTs stained for myogenin [8].

Flow cytometry can also be used to differentiate between IMT and ERMS as most cases of IMT are diploid with low S-phase fraction features which suggest benign lesions [3, 5] and ERMSs usually show DNA aneuploidy or diploidy with high proliferative fraction. The presence of DNA aneuploidy suggests the possibility of a malignancy.

A learning point for this case is that the rapid recurrence of the symptoms and regrowth of the prostate is inconsistent with the benign nature of IMT. To avoid misdiagnosis, a search for associated carcinoma should be taken. For our patient, after 2 surgeries for presumed benign prostatic hyperplasia, a histological diagnosis of pseudosarcomatous fibromyxoid tumour is a little surprising as it does not match the slow growing benign nature of the condition. A high index of suspicion is warranted here.

It may be difficult to differentiate these 2 disparate histological entities as presenting symptoms can be similar with gross haematuria being the most common symptom. Lower urinary symptoms are usually present as well. Inflammatory pseudotumors are benign, slow growing lesions that do not metastasize or undergo malignant transformation. Local recurrences are rarely observed after either endoscopic or open excision. In this case, the rapid recurrence of lower urinary tract symptoms after TURP is unusual and one must consider differential diagnoses of primary or metastatic urothelial carcinomas or sarcomatoid carcinoma of the bladder/prostate as well. Clinical context is essential in raising suspicion in order to make an accurate diagnosis.

It is believed that the best management for IMTs is complete surgical extirpation with preservation of normal tissues at the surgical margin [2, 9]. Other acceptable treatments are TURP, TURBT, or partial cystectomy. Because of the histologic similarity of inflammatory pseudotumors to certain malignant tumors, most authors recommend close surveillance [3].

As ERMSs are very rare in adults, most of the current knowledge is extrapolated from Intergroup Rhabdomyosarcoma Study (IRS) results which are case series from the paediatric population. The staging system used in IRS I–III has gained worldwide acceptance and is commonly used [10]. Adult genitourinary sarcomas account for only 2% of urological malignancies. For paediatric RMS, there are 3 histological variants, namely, botryoid, which consists of a cambium layer and is believed to confer a better prognosis in children, spindled variant, which is commonly found in scrotal specimens, and anaplastic variant, which is most commonly found in the head and neck regions. More than 90% of genitourinary tract RMSs are embryonal, mostly of the botryoid subtype [7]. Histologic subtyping of RMS has important prognostic and therapeutic ramifications as embryonal RMS, particularly the botryoid subtype, has a more favorable behavior than alveolar RMS.

In adults, bladder RMS may demonstrate alveolar or unclassified histology, commonly with anaplasia, and there is uniformly aggressive course. In the reported cases, the patients' age ranges from 23 to 85 years and none had associated CIS of the bladder. Morphologically, pure RMS in adult bladders consists of primitive undifferentiated round cells without obvious rhabdomyoblast differentiation and with varying degrees of anaplasia. Only 4 out of the 13 reported cases of RMS are of embryonal variant and the subtype heterogeneity of these 13 cases suggests that pure RMSs of adult bladders are likely different from the paediatric tumors [7]. For IRS I–III, anaplasia is seen only in 3.6% of the paediatric RMSs unlike those in adults. In adults, the presence of anaplasia confers a worse prognosis (45% 5-year survival versus 68% 5-year survival) and the unrelenting course of the disease is in stark contrast to the favorable outcome in children where the reported cure rate is 70–80% [11].

Although data is limited for adults, complete excision should be considered for localized disease as long as functional and/or cosmetic results are acceptable. Vulvar, vaginal, uterine, bladder, and prostatic RMSs in paediatric patients usually respond well enough to induction chemotherapy to render them locally resectable, often with clear resection margins [11]. In a retrospective analysis by Filipas, primary chemotherapy followed by radical surgery of RMS of the prostate and/or bladder in paediatric cases yielded excellent cure rates up to 80% [12].

Radiotherapy is also a major tool for the treatment of RMS, particularly for achieving local control in patients with residual microscopic or gross disease following surgery and chemotherapy [10]. Because ERMS of the bladder is rare, treatment options are not standardized. In Paner's series, 2 patients already had metastatic disease on diagnosis, 2 patients had adjuvant chemotherapy after transurethral bladder tumor resection, and 1 patient underwent radical cystectomy [7]. All 3 patients succumbed to the disease in less than a year from the time of diagnosis. There is only 1 adult patient with ERMS of the bladder in the reported literature that was treated with radiotherapy and survived with no evidence of disease after 30 months of follow-up [13]. The poor prognostic outcomes in these adult bladder RMSs mirror those adult RMSs of similar histology at other anatomic sites.

## 4. Conclusion

Rapid recurrence of lower urinary tract symptoms with rapid prostate regrowth after TURP is unusual and differential diagnoses of unusual bladder malignancies should be considered. A histological diagnosis of pseudosarcomatous fibromyxoid tumour should be viewed with caution if the clinical course of the disease is inconsistent.

## Abbreviations

RMS: Rhabdomyosarcoma
ERMS: Embryonal rhabdomyosarcoma
IMT: Inflammatory myofibroblastic tumour
PSFMT: Pseudosarcomatous fibromyxoid tumor
BPE: Benign prostate enlargement
TURP: Transurethral resection of prostate
IRS: Intergroup Rhabdomyosarcoma Study
BPH: Benign hyperplasia of the prostate.
